# New Halogenated Compounds from *Halimeda macroloba* Seaweed with Potential Inhibitory Activity against Malaria

**DOI:** 10.3390/molecules27175617

**Published:** 2022-08-31

**Authors:** Abeer H. Elmaidomy, Eman Maher Zahran, Raya Soltane, Ahlam Alasiri, Hani Saber, Che Julius Ngwa, Gabriele Pradel, Faisal Alsenani, Ahmed M. Sayed, Usama Ramadan Abdelmohsen

**Affiliations:** 1 Department of Pharmacognosy, Faculty of Pharmacy, Beni-Suef University, Beni-Suef 62511, Egypt; 2 Department of Pharmacognosy, Faculty of Pharmacy, Deraya University, 7 Universities Zone, New Minia 61111, Egypt; 3 Department of Basic Sciences, Adham University College, Umm Al-Qura University, Makkah 21955, Saudi Arabia; 4 Department of Biology, Faculty of Sciences, Tunis El Manar University, Tunis 1068, Tunisia; 5 Department of Botany and Microbiology, Faculty of Science, South Valley University, Qena 83523, Egypt; 6 Division of Cellular and Applied Infection Biology, Institute of Zoology, RWTH Aachen University, 52056 Aachen, Germany; 7 Department of Pharmacognosy, College of Pharmacy, Umm Al-Qura University, Makkah 21955, Saudi Arabia; 8 Department of Pharmacognosy, Faculty of Pharmacy, Nahda University, Beni-Suef 62513, Egypt; 9 Department of Pharmacognosy, Faculty of Pharmacy, Minia University, Minia 61519, Egypt

**Keywords:** malaria, *Plasmodium falciparum*, *Halimeda macrolaba*, cytochrome C, docking, molecular dynamics

## Abstract

Malaria is one of the most important infectious diseases worldwide. The causative of the most severe forms of malaria, *Plasmodium falciparum*, has developed resistances against all the available antimalarial drugs. In the present study, the phytochemical investigation of the green seaweed *Halimeda macroloba* has afforded two new compounds **1**–**2**, along with 4 known ones **3**–**6**. The structures of the compounds had been confirmed using 1& 2D-NMR and HRESIMS analyses. Extensive machine-learning-supported virtual-screening suggested cytochrome-C enzyme as a potential target for compound **2**. Docking, absolute-binding-free-energy (Δ*G*_binding_) and molecular-dynamics-simulation (MDS) of compound **2** revealed the strong binding interaction of this compound with cytochrome-C. In vitro testing for crude extract and isolated compounds revealed the potential in vitro inhibitory activity of both extract and compound **2** against *P. falciparum*. The crude extract was able to inhibit the parasite growth with an IC_50_ value of 1.8 ± 0.35 µg/mL. Compound **2** also showed good inhibitory activity with an IC_50_ value of 3.2 ± 0.23 µg/mL. Meanwhile, compound **6** showed moderate inhibitory activity with an IC_50_ value of 19.3 ± 0.51 µg/mL. Accordingly, the scaffold of compound **2** can be considered as a good lead compound for the future development of new antimalarial agents.

## 1. Introduction

Infectious diseases impose a significant burden on global public health and economic stability [1]. Malaria is a potentially life-threatening parasitic disease caused by *Plasmodium* protozoa and accounting for approximately 229 million cases and 409,000 fatalities in 2019 [2]. Currently, two antimalarial drugs are used to control infection: artemisinin, obtained from *Artemisia annua* L., and quinine, obtained from *Cinchona* sp. [2]. The emergence of resistance on the part of mosquitoes to these antimalarial drugs, the weak development of new antimalarial drugs, the logistical problems of these drugs in poor malaria-endemic countries, and the lack of efficient and safe vaccines might increase the complications of malaria in the future [3]. Accordingly, malaria will continue to present challenges to the global goal of its control and eradication, which makes searching for effective, safe, and affordable alternatives an urgent necessity.

Antimalarial drug discovery from marine sources is currently targeted due to reports of such sources yielding effective and safe antimalarial phytochemicals owing to variations in structure and biological activity [4]. The imidazole alkaloid, paenidigyamycin A isolated from *Ghanaian Paenibacillus* polymyxa strain De2sh, inhibited *P. falciparum* 3D7 with IC_50_  =  36 nmol/L [5]. The bromo-pyrrole alkaloids isolated from *Tedania braziliensis*, a marine sponge, are made up of new pseudo-ceratidine and its derivatives and exhibited potent activity against sensitive (3D7 strain) and resistant (K1 strain) *P. falciparum* strains (0.96–1.24, 5.11–6.49, and 3–6 μmol/L) [6]. Two strains of *P. falciparum* (drug-resistant K1 and drug-sensitive FCR3) were inhibited also by bromo-tyrosine alkaloid, ceratinadin E (1.03 and 0.77 μg/mL respectively), and psammaplysin F (3.77 and 2.45 μg/mL respectively), produced by Okinawan marine sponge (*Pseudoceratina* sp.) [7]. From an Australian bryozoan, *Orthoscuticella ventricose*; two new orthoscuticellines, A and B; three new *β*-carboline alkaloids, orthoscuticellines C−E; and six other known compounds were isolated. These compounds showed moderate activity against the same parasite (12–21 μmol/L) [8]. A new linear diterpene, bifurcatriol, was isolated from an Irish brown alga, *Bifurcaria bifurcate*, and inhibited drug-resistant *P. falciparum* K1 at low concentration (0.65 µg/mL) [9]. Three potent antimalarial sesquiterpenoids, smenotronic acid, ilimaquinone, and pelorol, isolated from the sponge *Hyrtios erectus*, showed promising in vitro activity against chloroquine-resistant *P. falciparum* strain Dd2 at IC_50_ 3.51, 2.11, and 0.8 µmol/L, respectively [10]. In in vitro antimalarial activity against *P. falciparum* 3D7, endoperoxide, sinuketal (isolated from soft corals *Sinularia* sp.), showed mild activity (IC_50_  =  80 µmol/L) [11]. Hoshinoamides A and B (isolated from cyanobacterium, *Caldora penicillata*) had moderate inhibitory activity against *P. falciparum* at an IC_50_ value of 0.52 and 1.0 μmol/L, respectively [12]. a new cyclic peptide, kakeromamide B, along with ulongamide A and lyngbyabellin A, isolated from the marine cyanobacterium Moorea producens collected from reef slopes off the shores of Tuvuca Island in Fiji, exhibited antiparasitic activity against *P. falciparum* blood-stages (EC_50_  =  8.9, 0.99 and 1.5 µmol/L, respectively) [13]. A macrolide with a 40-membered ring, palstimolide A, isolated from Central Pacific Ocean *Cyanobacterium*, exhibited interesting antiparasitic activities against the blood-stage of the *P. falciparum* Dd2 (IC_50_  =  223 nmol/L) [14]. Likewise, another new 24-membered polyhydroxy macrolide, bastimolide B, isolated along with a known bastimolide A from a cyanobacterium, *Okeania hirsute*, exhibited strong anti-plasmodial activity against chloroquine-sensitive *P. falciparum* strain HB3 (IC_50_  =  5.7  ±  0.7 μmol/L) [15]. Cholesterol and a new antimalarial mono-hydroxy acetylated sterol derivative, halymeniaol, were isolated from the marine alga *Halymenia floresii*. The former exhibited inhibitory activity against the chloroquine-resistant *P. falciparum* 3D7 strain (IC_50_  =  3.0 μmol/L) [16]. Sterols, kaimanol and saringosterol, with significant antimalarial activity against *P. falciparum* 3D7 strains (IC_50_  =  359 and 0.250 nmol/L respectively) were isolated from an Indonesian marine sponge, *Xestospongia* sp. *n*-hexane extract [17].

Marine plants, though rich in secondary metabolites with variant bioactivities, do not have a remarkable history of use in traditional medicine [18]. However, recent improvements in marine biology and engineering have aided in the investigation and scientific exploration of marine algae to identify and isolate novel compounds [18]. Marine seaweeds live in an environment with uneven circumstances of temperature, light, salinity, nutrients, and contaminants [19]. Therefore, they adapt to changing environmental conditions via production of a wide range of metabolites not found in organisms from terrestrial environments [19]. Hence, compounds extracted from marine seaweeds are an important source for the production of new medicines.

*Halimeda macroloba* is a widespread green seaweed in wide marine habitats which is always associated with coral reefs and therefore contains high amounts of calcium carbonate, causing it to be classified as a calcified or calcareous algae [20]. *Halimeda* attains a diversity of secondary metabolites which are still not fully explored, with few reported compounds which proved activity against human, fish, and shrimp pathogenic bacteria [21]. *Halimeda macroloba* generally grows in complex environmental conditions (relatively high salinity of seawater, high heavy metal content, and much susceptibility to surrounding organisms) [21]. This green seaweed proved antioxidant, antibacterial, and cytotoxic efficacies but has never been investigated against malaria, while other related species such as *H. gracilis* were [22]. In silico techniques including machine learning and artificial intelligence have significantly advanced during the last 15 years, to the point where they have become an integrated tool in the discovery and development of new therapeutics. These techniques have also facilitated the investigation of natural crude extracts to find out the potential bioactive chemical entity’s complex matrices [3,23].

Consequently, we aimed in the present work to explore the phytochemical profile of the green seaweed *Halimeda macroloba* and test its possible anti-plasmodial potential via virtual screening and physics-based molecular simulation.

## 2. Results and Discussion

### 2.1. Phytochemical Investigation of Halimeda macroloba Seaweed

**Figure 1:**
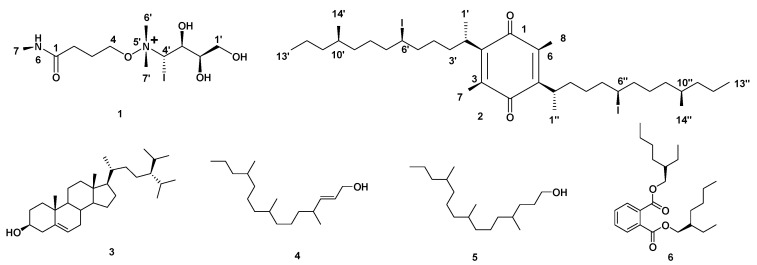
Structures of compounds isolated from *Halimeda macroloba* algae.

Based on the physicochemical and chromatographic properties, the spectral analyses from UV, ^1^H, and DEPT-Q NMR, as well as comparisons with the literature and some authentic samples, the crude ethanolic extract of *Halimeda macroloba* offered two new compounds, 4-*O*-(4‣-(dimethylamino)-4‣-iodobutan-5‣-yl-1‣,2‣,3‣-triol)-N-methylbutanamide **1**, and 2,5-bis(6-iodo-10-methyltridecan-2-yl)-3,6-dimethylcyclohexa-2,5-diene-1,4-dione **2**, along with four known compounds named as: 24-isopropyl cholesterol **3** [24], (*E*)-4,8,12-trimethylpentadec-2-en-1-ol **4** [25], 4,8,12-trimethylpentadecan-1-ol **5** [25], and bis(2-ethylhexyl) phthalate **6** [26] (Figure 1). Compound **3** was isolated here for the first time from the genus *Halimeda* (Figures S1–S14 and Figure 1).

**Figure 2:**
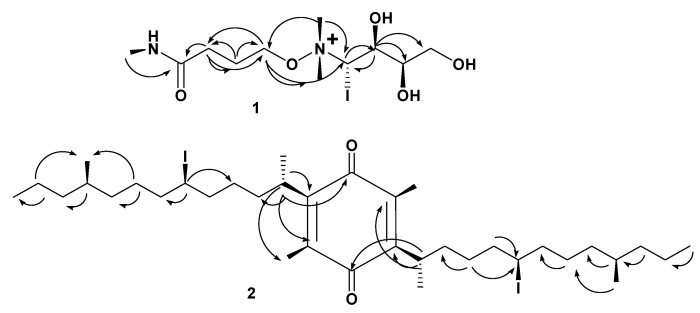
Selected HMBC (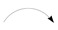) correlations of compounds **1, 2**.

Compound **1** (Table 1, Figure 1, see Supplementary Figures S1–S4) was obtained as a yellow amorphous solid. The HRESIMS data for compound **1** showed an adduct pseudo-molecular ion peak at *m*/*z* 392.0809 [M + H]^+^, consistent with the molecular formula C_11_H_25_IN_2_O_5_ and suggesting one degree of unsaturation. The IR spectrum suggested the presence of one iodide atom at 540 cm^−1^. The 1H, DEPT-Q, HSQC, and HMBC NMR data (Table 1, Supplementary Figures S1–S4, Figure 1 and Figure 2), showed five characteristic resonances: two methylene groups at *δ*_H_ 2.38 (2H, *br. s*) *δ*_C_ 34.0, *δ*_H_ 2.11 (2H, *br. s*) *δ*_C_ 20.3, one oxy-methylene group at *δ*_H_ 3.45 (2H, *m*) *δ*_C_ 67.1, with one quaternary carbon at *δ*_C_ 180.8 and one downfield methyl group at *δ*_H_ 2.00 (3H, *s*) *δ*_C_ 24.2, suggesting the characteristic structure of the 4-hydroxy-N-methylbutanamide unit [27].

Moreover, the 1H, DEPT-Q, HSQC, and HMBC NMR data (Table 1, Supplementary Figures S1–S4, Figure 1 and Figure 2) showed six characteristic resonances: one oxy-methylene group at *δ*_H_ 3.76, 3.86 (2H, *overlapped*) *δ*_C_ 64.3, two oxy-methine groups at *δ*_H_ 3.80 (1H, *overlapped*) *δ*_C_ 72.1, *δ*_H_ 3.91 (1H, *overlapped*) *δ*_C_ 70.6, one iodo-methine group at *δ*_H_ 3.37 (1H, *overlapped*) *δ*_C_ 54.2, and two methyl groups at *δ*_H_ 3.28 (6H, *s*) *δ*_C_ 53.7, 53.7, suggesting the characteristic structure of the 4-(dimethylamino)butane-1,2,3-triol [28]. Additionally, the HMBC experiment showed that the characteristic ^4^*J* HMBC correlation of proton H-4 *δ*_H_ 3.45 (2H, *m*) with C-6′, 7′ *δ*_C_ 53.7 confirmed the attachment of the 4-hydroxy-N-methylbutanamide unit of C-4 at N-5′ of the 4-(dimethylamino)-butane-1,2,3-triol unit (Figure 2). The NMR data for C-4′ at *δ*_H_ 3.37 (1H, *overlapped*) *δ*_C_ 54.2 suggested the attachment of iodide atom at C-4′. The relative stereochemistry of compound **1** was deduced using the *J* values and a Nuclear Overhauser Effect (NOE) experiment (Table 1). The NOEs observed between H-7, H-2′, H-3′, H-6′, H-7′ suggested that they are in the same plan, while the NOEs observed between H-4′ suggested they are on the other side of the compound skeleton. Accordingly, compound **1** was identified as 4-*O*-(4’-(dimethylamino)-4’-iodobutan-5’-yl-1’,2’,3’-triol)-N-methylbutanamide.

Compound **2** (Table 2, Figure 1, see Supplementary Figures S5 and S6) was obtained as a white amorphous solid. The HRESIMS data for compound **2** showed an adduct pseudo-molecular ion peak at *m*/*z* 781.2919 [M + H]^+^, consistent with the molecular formula C_36_H_63_I_2_O_2_ and suggesting 5 degrees of unsaturation. The IR spectrum suggested the presence of an iodide atom at 540 cm^−1^. The 1H, DEPT-Q, HSQC, and HMBC NMR data (Table 2, Supplementary Figures S5 and S6, Figure 1 and Figure 2) showed eight characteristic resonances: six quaternary carbons at *δ*_C_ 187.8, 188.2, 145.0, 141.1, 141.0, and 140.7, and two methyl groups at *δ*_H_ 2.03, 2.04 (6H, *s*), and *δ*_C_ 12.5, suggesting the characteristic structure of the 3,6-dimethyl,1,4- benzoquinone unit [29]. The 1H, DEPT-Q, HSQC, and HMBC NMR data (Table 2, Supplementary Figures S5 and S6, Figure 1 and Figure 2) showed 28 characteristic resonances: six methyl groups at *δ*_H_ 1.27, 1.27 (6H, *d*, *J* = 4.2) *δ*_C_ 20.2, 20.3, *δ*_H_ 0.88, 0.87 (6H, *d*, *J* = 4.7) *δ*_C_ 12.8, 12.9, and *δ*_H_ 1.27, 1.26 (6H, *t*, *J* = 6.0, 6.1) *δ*_C_ 23.2, 23.2; sixteen methylene groups at *δ*_H_ 1.10–1.32 (4H, *overlapped*) *δ*_C_ 37.8, 38.0, *δ*_H_ 1.10–1.32 (4H, *overlapped*) *δ*_C_ 25.3, 25.3, *δ*_H_ 1.49–1.69 (4H, *overlapped*) *δ*_C_ 30.1, 30.2, *δ*_H_ 1.49–1.69 (4H, *overlapped*) *δ*_C_ 30.2, 30.2, *δ*_H_ 1.10–1.32 (4H, *overlapped*) *δ*_C_ 25.0, 25.0, *δ*_H_ 1.10–1.32 (4H, *overlapped*) *δ*_C_ 39.9, 39.9, *δ*_H_ 1.10–1.32 (4H, *overlapped*) *δ*_C_ 40.8, 40.8, *δ*_H_ 1.10–1.32 (4H, *overlapped*) *δ*_C_ 21.9, 22.0; and six methine groups at *δ*_H_ 2.5 (2H, *overlapped*) *δ*_C_ 28.5, 28.5, *δ*_H_ 3.66 (2H, *m*) *δ*_C_ 33.3, 33.4, *δ*_H_ 1.49–1.69 (2H, *overlapped*) *δ*_C_ 27.1, and 27.1, suggesting the characteristic structure of two units of 4-methyltridecane [16]. Additionally, the HMBC experiment showed the characteristic ^2^*J* HMBC correlation of proton H-2′, 2″ *δ*_H_ 2.50 (2H, *overlapped*) with C-2, 5 *δ*_C_ 145.0, 141.1, and confirmed the attachment of the two 4-methyltridecane units C-2′, C-2″ at C-2, C-5 of 3,6-dimethyl,1,4- benzoquinone unit (Figure 2). The NMR data for C-6′, 6″ at *δ*_H_ 3.66 (2H, *overlapped*) *δ*_C_ 33.3, 33.4 suggested the attachment of iodide atoms at C-6′, 6″. The NOEs observed between H-1′, H-1″ suggested that they are in the same plan, while the NOEs observed between H-6′, H-14′, H-6″, and H-14″ suggested they are on the other side of the compound skeleton. Accordingly, compound **2** was identified as 2,5-bis(6-iodo-10-methyltridecan-2-yl)-3,6-dimethylcyclohexa-2,5-diene-1,4-dione, which is similar to menzoquinone (isolated from *Antarctic* macroalgae) in structure, but different from the additional methyl and saturated 4-methyltridecane units at C-3 and 5, respectively [29].

### 2.2. Predicting Possible Biological Activity

In order to predict the probable biological activity of the isolated compounds, we subjected their modeled structures to a machine learning-based virtual screening platform called Prediction of Activity Spectra of Substances (PASS; http://www.way2drug.com/passonline/products.php, accessed on 16 August 2022). This platform utilizes a pharmacophore-based screening algorithm to score the biological activities of query structure. Possible active score (Pa) > 0.5 indicates high probability of being active in the corresponding biological activity category [30]. After submitting all structures of the isolated compounds, compound **2** showed an interesting Pa score (0.892) for the cytochrome-C reductase inhibitor. This enzyme is involved in many essential biological processes in many eukaryotes, particularly in malaria [31,32]. Accordingly, this preliminary in silico screening directs our attention to test both the crude extract of *H. macrolaba* and the isolated compounds against *P. falciparum* in vitro. The results indicated that the extract inhibits the replication of *P. falciparum* in a dose-dependent manner with an IC_50_ value of 1.8 ± 0.35 µg/mL. Moreover, compound **2** also showed good inhibitory activity with an IC_50_ value of 3.2 ± 0.23 µg/mL, while compound **6** showed moderate inhibitory activity with an IC_50_ value of 19.3 ± 0.51 µg/mL (Table 3).

### 2.3. Binding Mode Analysis and Absolute Binding Free Energy Calculation

According to the in vitro antimalarial inhibitory activity results, compound **2** is a potential antimalarial agent that probably targets and inhibits the parasite’s cytochrome-C reductase.

To study the binding mode of compound **2** with cytochrome-C reductase, the modeled structure of this compound was docked into the atovaquone-binding site of cytochrome bc1 reductase (PDB code: 4PD4; atovaquone is a quinone-based antimalarial drug) [33]. The resulting binding poses (10 poses) were almost identical with docking poses ranging from −10.83 to −9.94 kcal/mol (Table S1). Subsequently, these poses were subjected to molecular-dynamics simulation-based absolute binding free energy determination (Δ*G*_binding_) to assess the pose with the highest affinity toward the enzyme’s binding site using the free energy perturbation method (FEP) [34]. The second binding pose, with a docking score of −10.52 kcal/mol, was the best in terms of affinity to the binding site, where it attained the lowest Δ*G*_binding_ value of −8.33 kcal/mol. Regarding the co-crystalized inhibitor atovaquone, its modeled structure was re-docked into the enzyme active site, and similarly to compound **2**, ten poses for the docked structure were generated with docking scores ranging from −9.76 to −9.23 kcal/mol (Table S1). The Δ*G*_binding_ of these generated poses was also determined, where the first pose got the lowest value (−14.46 kcal/mol). The calculated RMSD of this pose with co-crystalized inhibitor atovaquone was 1.17 Å.

From the previous findings we can conclude that compound **2** has a comparable affinity with the co-crystalized ligand toward cytochrome-C reductase. Additionally, its lower affinity is comparable to the co-crystalized inhibitor, which might be attributed to its higher flexibility (i.e., higher number of rotatable bonds, **22** vs. **2** for atovaquone).

**Figure 3:**
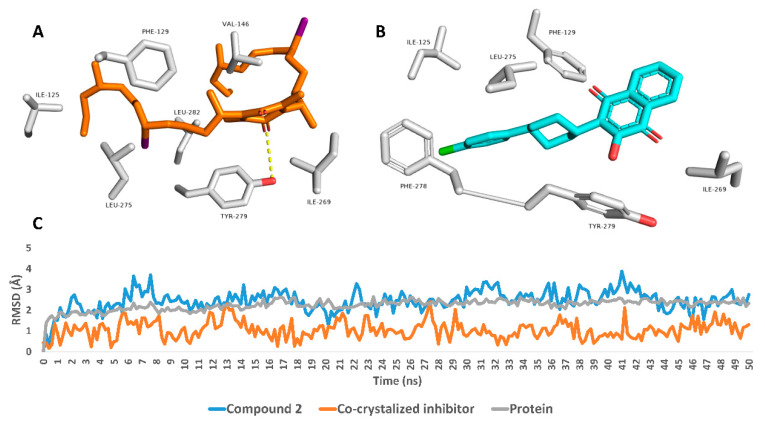
Binding modes of compound **2** and atovaquone inside the cytochrome bc1’s binding site (PDB ID: 4PD4) (**A** and **B**, respectively). These poses were extracted as the top-populated poses from 50 ns MDS experiments. **C**: RMSDs of compound **2** and atovaquone inside the cytochrome bc1’s binding site over the 50ns long MDS runs.

To study the dynamic binding mode of compound **2** and atovaquone inside the enzyme’s active site, their binding poses in terms of Δ*G*_binding_ were subjected to 50 ns long MDS. As shown in Figure 3, both structures achieved good binding stability over the course of simulation; however, atovaquone was more stable with an average RMSD of 1.34 Å (the average RMSD of compound **2** was 3.23 Å). The extracted top-populated binding poses of both structures revealed that both their binding and stability inside the binding pocket were achieved primarily via hydrophobic interactions. Atovaquone established hydrophobic interactions mainly with PHE-129, ILE-269, LEU-275, PHE-278, and TYR-279. Regarding compound **2**, its structure was larger, more flexible, and richer in hydrophobic hydrocarbon moieties and attained two hydrophobic iodine atoms. Hence, it was able to establish more hydrophobic interactions inside the binding pocket, e.g., ILE-125, PHE-129, VAL-146, ILE-269, LEU-275, TYR-279, and LEU-282, moreover establishing a prominent single H-bond with TYR-279. Taken together, compound **2** can be considered a good scaffold for the future development of new antimalarial agents targeting cytochrome-C. However, this scaffold (i.e., compound **2**) is not a drug-like molecule according to Lipinski’s and Veber’s rules of drug-likeness (Molecular weight > 500, LogP > 4.15, and the number of rotatable bonds > 10). The removal of one of the two long aliphatic sidechains can significantly improve the drugability of this scaffold. Accordingly, in our next stage of investigating this probable antimalarial molecule, its biological activity will be fully characterized and its essential structural features will be identified so that its structure can be modified synthetically to a drug-like molecule.

To the best of our knowledge, this is the first study investigating the phytochemical environment of *H. macrolaba* with a focus on its anti-plasmodium efficacy. The phytochemical analyses led to the identification of six compounds, with the predominance of aliphatic hydrocarbon moieties as well as strong antimalarial activity (IC_50_ 1.8 ± 0.354 µg/mL). Since bioactive extracts are categorized to be potent as antimalarials when their IC_50_ values are less than 10 µg/mL, *H. macrolaba* extract can be considered as a potential source of potent antimalarial agents [35]. Interestingly, a previous machine-learning study reported that the most persuasive physicochemical factor for antimalarial drugs to penetrate red blood cells is protein binding. The less a drug is bound to protein, the more it is freely available to penetrate the red blood cell. Drugs with aromatic hydrocarbons and/or aliphatic hydrocarbons may have a higher amount of freely available drug in the plasma to penetrate the red blood cells, facilitating their pharmacodynamic activities [36]. That might explain why compound **2** [2,5-bis (6-iodo-10-methyltridecan-2-yl)-3,6-dimethylcyclohexa-2,5-diene-1,4-dione], with its high number of aliphatic hydrocarbons, attained the highest binding score toward cytochrome c reductase. Moreover, this compound attains a quinone moiety that strengthens the antimalarial activity, which was supported by the reports of many quinone-derived compounds with potent antimalarial efficacies [37]. In a previous study, physcion and emodin (aromatic quinone derivatives) proved strong antimalarial activities with IC_50_ values of 0.9 and 1.9 µM, respectively [38]. Moreover, atovaquone is a well-known antimalarial quinone-based compound that is used in combination with proguanil (Malarone^®^) for the control of malaria infections worldwide [38]. Regarding compound **2**, a new compound, this is the first report of a quinone-based aliphatic compound with potent antimalarial efficacy. Accordingly, and in addition to recent related published data, machine learning could be a powerful approach to be widely used in various scientific fields for finding valuable information from data. The aims of a machine-learning model progression can be employed to build a robust predictive model, especially as most antimalarial drugs are still orphan [36] and data about their safety are limited. More importantly, the target prediction software has become an integral part of the drug discovery platform, which reduces the time and effort required for screening huge libraries of chemical compounds to find out available drug candidates. Such in silico tools could be employed in drug discovery from natural sources, with the ability to prioritize possibly active metabolites in a natural extract; hence, isolation and identification efforts will be directed toward top-scoring candidates.

## 3. Materials and Methods

### 3.1. Seaweed Samples Collection and Identification

Specimens of the green seaweed *Halimeda macroloba* were collected during May 2021 from the littoral zone of shorelines in Savage City on the Red Sea coast, Egypt. The collected seaweed samples were washed well with seawater, then with tap water, and finally with distilled water to remove any impurities, sand particles, and salts on their surfaces. The samples were kept in sterile clean plastic bottles and transported chilled in an ice box to the laboratory. The specimens were kindly identified according to standard taxonomic keys, and a voucher specimen (2021-BuPD 82) was deposited at the Department of Pharmacognosy, Faculty of Pharmacy, Beni-Suef University, Egypt.

### 3.2. Chemicals and Reagents

The solvents employed in this work included *n*-hexane (*n*-hex., boiling point b.p. 60–80 °C), dichloromethane (DCM), ethyl acetate (EtOAC), ethanol, and methanol (MeOH), all purchased from El-Nasr Company for Pharmaceuticals and Chemicals (Cairo, Egypt). Deuterated solvents used for chromatographic and spectroscopic analyses were purchased from Sigma-Aldrich (Saint Louis, MO, USA), including methanol-*d_4_* (CD_3_OD-*d_4_*), dimethyl sulfoxide-*d_6_* (DMSO-*d*_6_), and chloroform-*d* (CDCL_3_-*d*). Column chromatography (CC) was performed using silica gel 60 (63–200 μm, E. Merck, Sigma-Aldrich), and silica gel GF_254_ for thin-layer chromatography (TLC) (El-Nasr Company for Pharmaceuticals and Chemicals, Cairo, Egypt) was employed for vacuum liquid chromatography (VLC). Thin-layer chromatography (TLC) was carried out using pre-coated silica gel 60 GF_254_ plates (E. Merck, Darmstadt, Germany; 20 cm × 20 cm, 0.25 mm in thickness). Spots were visualized by spraying with dragendorff reagent (DRR) (solution a: 0.85 g basic bismuth nitrate in 10 mL glacial acetic acid and 40 mL water under heating, solution b: 8 g potassium iodide in 30 mL water, then mix a + b (1:1), then 1 ml of the mixture was mixed with 2 mL glacial acetic acid and 10 mL water) and para-anisaldehyde (PAA) reagent (85:5:10:0.5 absolute EtOH:sulfuric acid:G.A.A.:*para*-anisaldehyde), followed by heating at 110 °C [39].

### 3.3. Spectral Analyses

Proton ^1^H and Distortionless Enhancement by Polarization Transfer-Q (DEPT-Q) ^13^C NMR spectra were recorded at 400 and 100 MHz, respectively. Tetramethylsilane (TMS) was used as an internal standard in CDCL_3_-*d* and CD_3_OD-*d_4_*, utilizing residual solvent peaks (*δ*_H_ = 7.26; and *δ*_C_ 77.2) and (*δ*_H_ = 3.34; 4.78; and *δ*_C_ 49.9) as references, respectively. Measurements were performed on a Bruker Advance III 400 MHz with BBFO Smart Probe and a Bruker 400 MHz EON Nitrogen-Free Magnet (Bruker AG, Billerica, MA, USA). Carbon multiplicities were determined using a DEPT-Q experiment. The ultraviolet radiation (UV) spectrum in methanol was obtained using a Shimadzu UV 2401PC spectrophotometer (Shimadzu Corporation-UV-2401PC/UV-2501PC, Kyoto, Japan). Infrared (IR) spectra were measured using a Jasco FTIR 300E infrared spectrophotometer. HRESIMS data were obtained using an Acquity Ultra Performance Liquid Chromatography system coupled with a Synapt G2 HDMS quadrupole time-of-flight hybrid mass spectrometer (Waters, Milford, MA, USA).

### 3.4. Extraction and Fractionation of Halimeda Macroloba

The *Halimeda macroloba* samples (0.25 kg) were collected and air-dried in the shade for one month, dried, then finely powdered using an OC-60B/60B grinding machine (60–120 mesh, Henan, Mainland China) [40,41]. The powder was extracted by maceration using 70% ethanol (5 L, 3×, seven days each) at room temperature and concentrated under vacuum at 45 °C using a rotary evaporator (Buchi Rotavapor R-300, Cole-Parmer, Vernon Hills, IL, USA) to afford 50 g crude extract [42,43]. The dry extract was suspended in 100 mL distilled water (H_2_O), and successively portioned with solvents of different polarities (*n*-Hex., DCM). The organic phase in each step was separately evaporated under reduced pressure to afford the corresponding fractions I (5.0 g) and II (25.0 g), respectively, while the remaining mother liquor was then concentrated down to give the aqueous fraction (20.0 g). All the resulting fractions were kept at 4 °C for biological and phytochemical investigations [44,45].

### 3.5. Isolation and Purification of Compounds

Fractions I and II (30 g) were gathered according to TLC, then subjected to VLC fractionation using silica gel GF_254_ (column 6 cm × 30 cm, 100 g). Elution was performed using DCM:EtoAc gradient mixtures in order of increasing polarities (0–20%, 1%, 250 mL each). The effluents from the column were collected in fractions (250 mL each), and each fraction was concentrated and monitored by TLC using the system DCM:EtoAc 9.5:0.5 and PAA reagent. Similar fractions were grouped and concentrated to provide five sub-fractions (I_1_–I_5_); each one was further purified on a Sephadex LH_20_ column (0.25–0.1 mm, 100 cm × 0.5 cm, 100 gm) and eluted with MeOH to afford compound **2** (20 mg), **3** (7 mg), **4** (10 mg), **5** (7 mg), and **6** (10 mg). Finally, the liquid fraction (5 g) was further purified on a Sephadex LH_20_ column (0.25–0.1 mm, 100 cm × 0.5 cm, 100 gm), where it was eluted with MeOH to afford compound **1** (2 g).

**4-*O*-**(**4′-**(**dimethylamino**)**-4′-iodobutan-5′-yl-1′,2′,3′-triol**)**-N-methylbutanamide** (**1**)**:** yellow powder; [UV (MeOH) *λ*_max_ (log_ε_) 280 (5.5), 270 (6.0), 300 (4.5) nm; IR υ_max_ (KBr) 3600, 3500, 3400, 3100, 1640, 1550, 1350, 1300, 1000, 540 cm^−1^; NMR data; see Table 1; HRESIMS *m*/*z* 392.0809 [M + H]^+^ (calc. for C_11_H_25_IN_2_O_5_, 392.0808).

**2,5-bis**(**6-iodo-10-methyltridecan-2-yl**)**-3,6-dimethylcyclohexa-2,5-diene-1,4-dione** (**2**)**:** white powder; [UV (MeOH) *λ*_max_ (log_ε_) 282 (5.5), 273 (6.0), 306 (4.5) nm; IR υ_max_ (KBr) 3100, 1720, 1670, 1465, 540 cm^−1^; NMR data; see Table 2; HRESIMS *m*/*z* 781.2919 [M + H]^+^ (calc. for C_36_H_63_I_2_O_2_, 781.2917).

### 3.6. Antimalarial Assay

The anti-plasmodial effect of the seaweed extract on *P. falciparum* erythrocytic replication in vitro was investigated using the Malstat assay [3]. The details of this method can be found in the supplementary file.

### 3.7. Biological Activity Prediction

Prediction of the probable biological activities of the isolated compounds was carried out using the (www.way2drug.com, accessed on 16 August 2022) [46]. The full details of this virtual screening method are provided in the supplementary file.

### 3.8. Molecular Docking, Δg_binding_ Calculation, and Molecular Dynamics Simulation

Docking, Δ*G*_binding_ calculation, and molecular dynamics simulation experiments were carried out as previously described [47]. The detailed methods can be found in the supplementary file.

## 4. Conclusions

Phytochemical investigation of the green seaweed *Halimeda macroloba* led to the isolation of two new compounds and four previously reported ones. The compounds’ structures were confirmed using 1D, 2D NMR, and HRESIMS analyses. The cytochrome-C enzyme, the critical target for the malaria parasite, was identified as a possible target for compound **2** after a machine learning-based virtual screening. The mode of interaction of compound **2′**s scaffold inside the active site of cytochrome-C was putatively determined using comprehensive molecular docking and MDS experiments. In addition, compound **2′**s affinity to the active site was calculated in terms of absolute binding free energy (Δ*G*_binding_ = −14.46 kcal/mol). In vitro against *P. falciparum* showed the potential inhibitory activity of compound **2** against the parasite. Accordingly, compound **2**’s scaffold can be considered as a promising lead compound for future antimalarial drug development.

## Figures and Tables

**Table 1:** DEPT-Q (400 MHz) and ^1^H (100 MHz) NMR data of compounds **1** in CD_3_OD-*d_4_*; Carbon multiplicities were determined by the DEPT-Q experiments.

	*^δ^*_C_	*^δ^*_H_ (*J* in Hz)
1	180.8, qC	
2	34.0, CH_2_	2.38, *br. s*
3	20.3, CH_2_	2.11, *br. s*
4	67.1, CH_2_	3.45, *m*
5		
6		
7	24.2, CH_3_	2.00, *s*
1′	64.3, CH_2_	3.76, 3.86, *overlapped*
2′	72.1, CH	3.80, *overlapped*
3′	70.6, CH	3.91, *overlapped*
4′	54.2, CH	3.37, *overlapped*
5′		
6′, 7′	53.7, 53.7, CH_3_	3.28, *s*

qC, quaternary, CH, methine, CH_2_, methylene, CH_3_, methyl carbons.

**Table 2:** DEPT-Q (400 MHz) and ^1^H (100 MHz) NMR data of compounds **2** in CDCl_3_-*d*; carbon multiplicities were determined by the DEPT-Q experiments.

Position	*^δ^*_C_	*^δ^*_H_ (*J* in Hz)
1	187.8, qC	
2	145.0, qC	
3	140.7, qC	
4	188.2, qC	
5	141.1, qC	
6	141.0, qC	
7,8	12.5, 12.5, CH_3_	2.03, 2.04, *s*
1′, 1″	20.2, 20.3, CH_3_	1.27, *d* (4.2)
2′, 2″	28.5, 28.5, CH	2.50, *overlapped*
3′, 3″	37.8, 38.0, CH_2_	1.10–1.32, *overlapped*
4′, 4″	25.3, 25.3, CH_2_	1.10–1.32, *overlapped*
5′, 5″	30.1, 30.2, CH_2_	1.49–1.69, *overlapped*
6′, 6″	33.3, 33.4, CH	3.66, *m*
7′, 7″	30.2, 30.2, CH_2_	1.49–1.69, *overlapped*
8′, 8″	25.0, 25.0, CH_2_	1.10–1.32, *overlapped*
9′, 9″	39.9, 39.9, CH_2_	1.10–1.32, *overlapped*
10′, 10″	27.1, 27.1, CH	1.49–1.69, *overlapped*
11′, 11″	40.8, 40.8, CH_2_	1.10–1.32, *overlapped*
12′, 12′’	21.9, 22.0, CH_2_	1.10–1.32, *overlapped*
13′, 13″	12.8, 12.9, CH_3_	0.88, 0.87, *d* (4.7)
14′, 14″	23.2, 23.2, CH_3_	1.27, 1.26, *t* (6.0, 6.1)

qC, quaternary, CH, methine, CH_2_, methylene, CH_3_, methyl carbons.

**Table 3:** Antimalarial activity of *H. macrolaba* extract and its isolated compounds **1–6** expressed as IC_50_ values.

Compound	IC_50_ (µg/mL)
1	>50
2	3.2 ± 0.23
3	>50
4	>50
5	>50
6	19.3 ± 0.51
Crude extract	1.8 ± 0.35
Chloroquine	0.022 ± 0.018

## Data Availability

Not applicable.

## Supplementary Materials

The following are available online at https://www.mdpi.com/article/10.3390/molecules27175617/s1, Figure S1: 1H NMR spectrum of compound **1** measured in CD3OD-d4 at 400 MHz; Figure S2: DEPT-Q NMR spectrum of compound **1** measured in CD3OD-d4 at 100 MHz, Figure S3: HSQC spectrum of compound **1** measured in CD3OD-d4; Figure S4: HMBC spectrum of compound **1** measured in CD3OD-d4; Figure S5: 1H NMR spectrum of compound **2** measured in CDCL3-d at 400 MHz; Figure S6: DEPT-Q NMR spectrum of compound **2** measured in CDCL3-d at 100 MHz; Figure S7: 1H NMR spectrum of compound **3** measured in CDCL3-d at 400 MHz; Figure S8: DEPT-Q NMR spectrum of compound **3** measured in CDCL3-d at 100 MHz; Figure S9: 1H NMR spectrum of compound **4** measured in CDCL3-d at 400 MHz; Figure S10: DEPT-Q NMR spectrum of compound **4** measured in CDCL3-d at 100 MHz; Figure S11: 1H NMR spectrum of compound **5** measured in CDCL3-d at 400 MHz; Figure S12: DEPT-Q NMR spectrum of compound **5** measured in CDCL3-d at 100 MHz; Figure S13: 1H NMR spectrum of compound **5** measured in CDCL3-d at 400 MHz; Figure S14: DEPT-Q NMR spectrum of compound **5** measured in CDCL3-d at 100 MHz. References [48,49,50,51,52,53,54] are cited in the Supplementary Materials.
