# Supplementary material for: New Halogenated Compounds from Halimeda macroloba Seaweed with Potential Inhibitory Activity against Malaria
Source: Molecules. 2022 Aug 31;27(17):5617. doi: 10.3390/molecules27175617 (PMC9457719; doi:10.3390/molecules27175617)
Supplement: Supplementary file 1 [file molecules-27-05617-s001.zip › molecules-1890774-supplementary.pdf]

New Halogenated Compounds from *Halimeda Macroloba* Seaweed with Potential Inhibitory Activity against Malaria

Abeer H. Elmaidomy [1,†], Eman Maher Zahran [2,†], Raya Soltane [3,4], Ahlam Alasiri [3], Hani Saber [5], Che Julius Ngwa [6], Gabriele Pradel [6], Faisal Alsenani [7], Ahmed M. Sayed [8,*] and Usama Ramadan Abdelmohsen [2,9,*]

[1] Department of Pharmacognosy, Faculty of Pharmacy, Beni-Suef University, Beni-Suef 62511, Egypt
[2] Department of Pharmacognosy, Faculty of Pharmacy, Deraya University, 7 Universities Zone, New Minia 61111, Egypt
[3] Department of Basic Sciences, Adham University College, Umm Al-Qura University, Makkah 21955, Saudi Arabia
[4] Department of Biology, Faculty of Sciences, Tunis El Manar University, Tunis 1068, Tunisia
[5] Department of Botany and Microbiology, Faculty of Science, South Valley University, Qena 83523, Egypt
[6] Division of Cellular and Applied Infection Biology, Institute of Zoology, RWTH Aachen University, 52056 Aachen, Germany
[7] Department of Pharmacognosy, College of Pharmacy, Umm Al-Qura University, Makkah 21955, Saudi Arabia
[8] Department of Pharmacognosy, Faculty of Pharmacy, Nahda University, Beni-Suef 62513, Egypt
[9] Department of Pharmacognosy, Faculty of Pharmacy, Minia University, Minia 61519, Egypt
* Correspondence: ahmed.mohamed.sayed@nub.edu.eg (A.M.S.); usama.ramadan@mu.edu.eg (U.R.A.)
† These authors contributed equally to this work.

List of Figures Contents

Figure S1. 1H NMR spectrum of compound **1** measured in CD_3OD-*d_4* at 400 MHz

Figure S2. DEPT-Q NMR spectrum of compound **1** measured in CD_3OD-*d_4* at 100 MHz

Figure S3. HSQC spectrum of compound **1** measured in CD_3OD-*d_4*

Figure S4. HMBC spectrum of compound **1** measured in CD_3OD-*d_4*

Figure S5. 1H NMR spectrum of compound **2** measured in $CDCL_3$-*d* at 400 MHz

Figure S6. DEPT-Q NMR spectrum of compound **2** measured in $CDCL_3$-*d* at 100 MHz

Figure S7. 1H NMR spectrum of compound **3** measured in $CDCL_3$-*d* at 400 MHz

Figure S8. DEPT-Q NMR spectrum of compound **3** measured in $CDCL_3$-*d* at 100 MHz

Figure S9. 1H NMR spectrum of compound **4** measured in $CDCL_3$-*d* at 400 MHz

Figure S10. DEPT-Q NMR spectrum of compound **4** measured in $CDCL_3$-*d* at 100 MHz

Figure S11. 1H NMR spectrum of compound **5** measured in $CDCL_3$-*d* at 400 MHz

Figure S12. DEPT-Q NMR spectrum of compound **5** measured in $CDCL_3$-*d* at 100 MHz

Figure S13. 1H NMR spectrum of compound 5 measured in $CDCL_3$-*d* at 400 MHz

Figure S14. DEPT-Q NMR spectrum of compound 5 measured in $CDCL_3$-*d* at 100 MHz

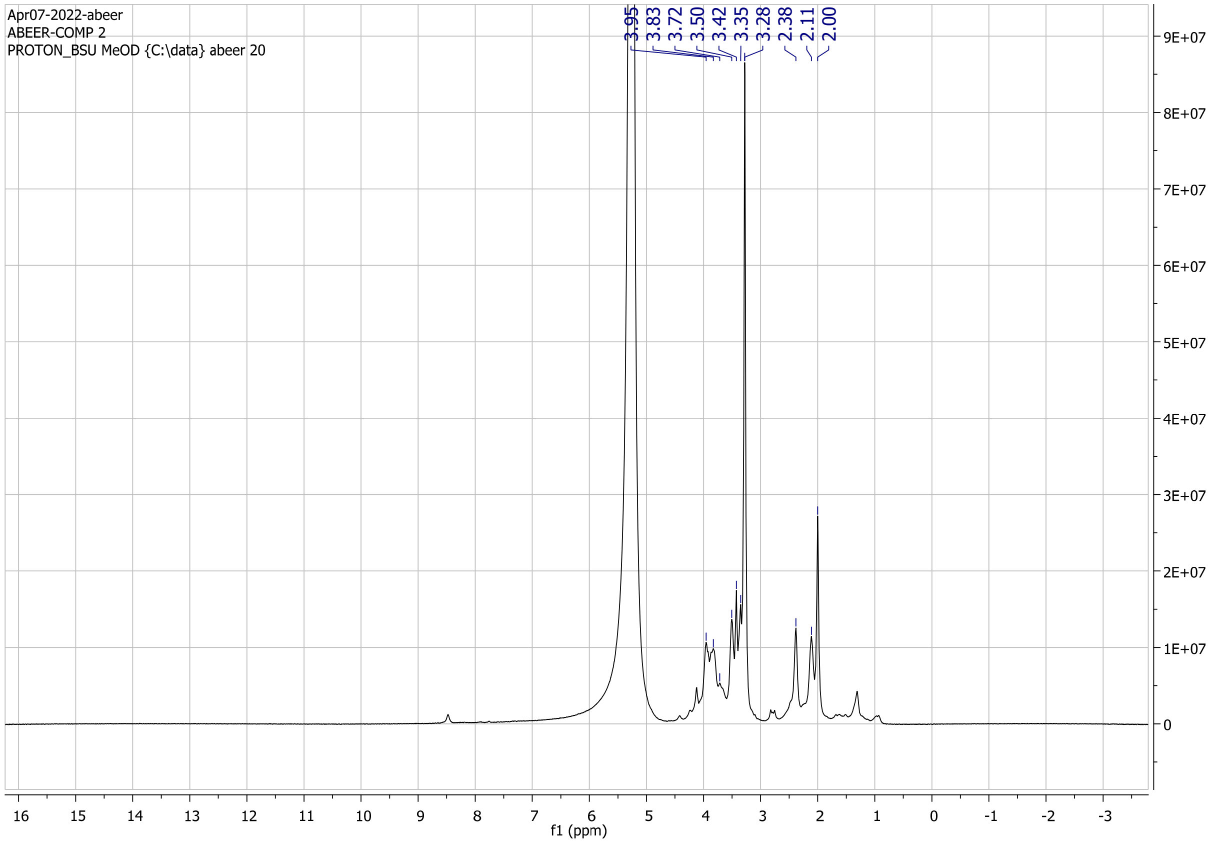

Figure S1. 1H NMR spectrum of compound **1** measured in CD_3OD-d_4 at 400 MHz

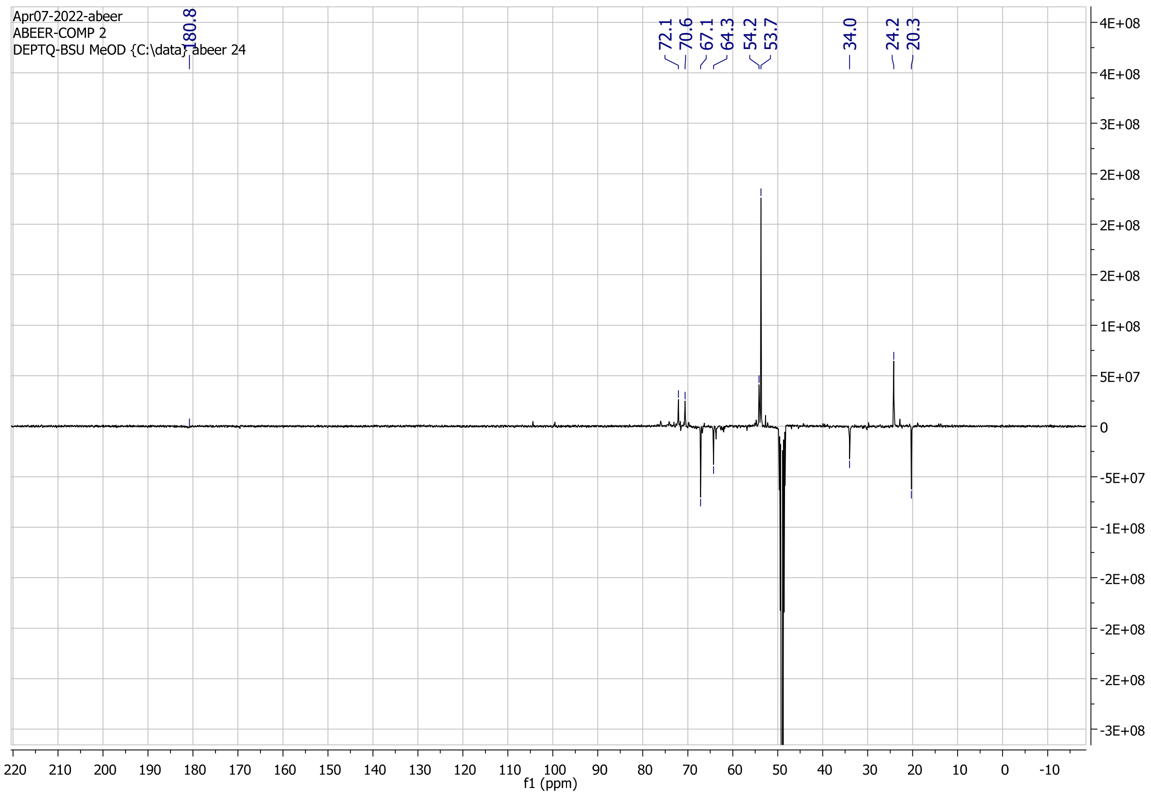

Figure S2. DEPT-Q NMR spectrum of compound **1** measured in CD_3OD-d_4 at 100 MHz

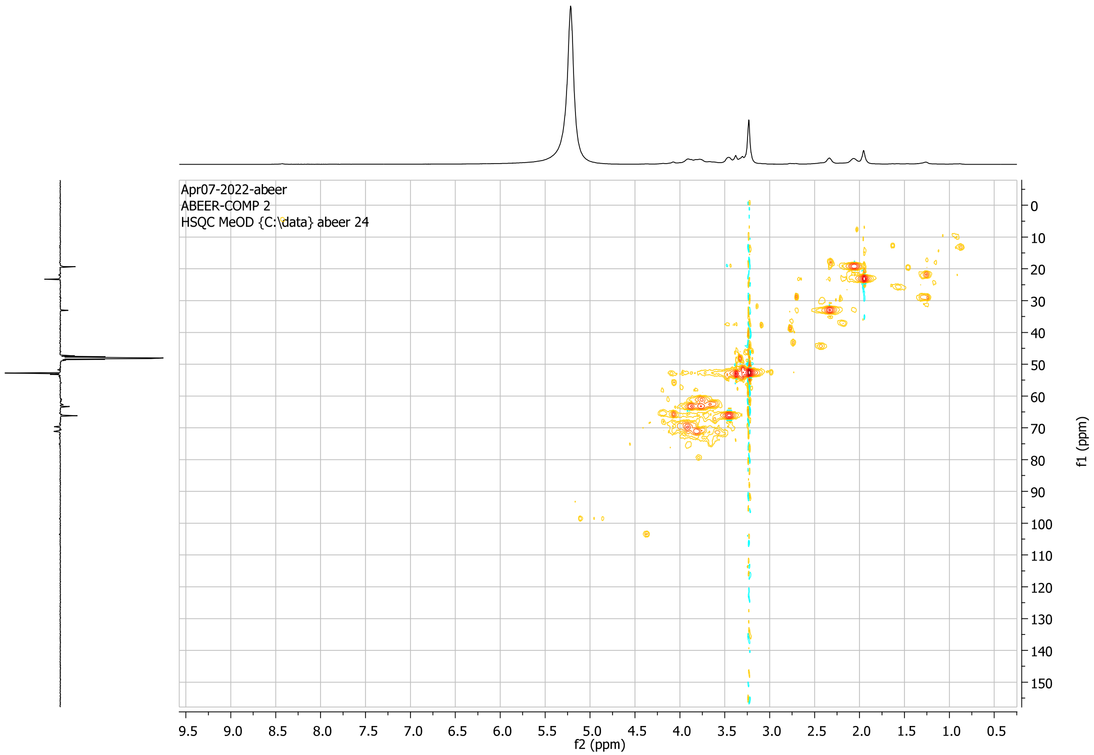

Figure S3. HSQC spectrum of compound **1** measured in CD_3OD-d_4

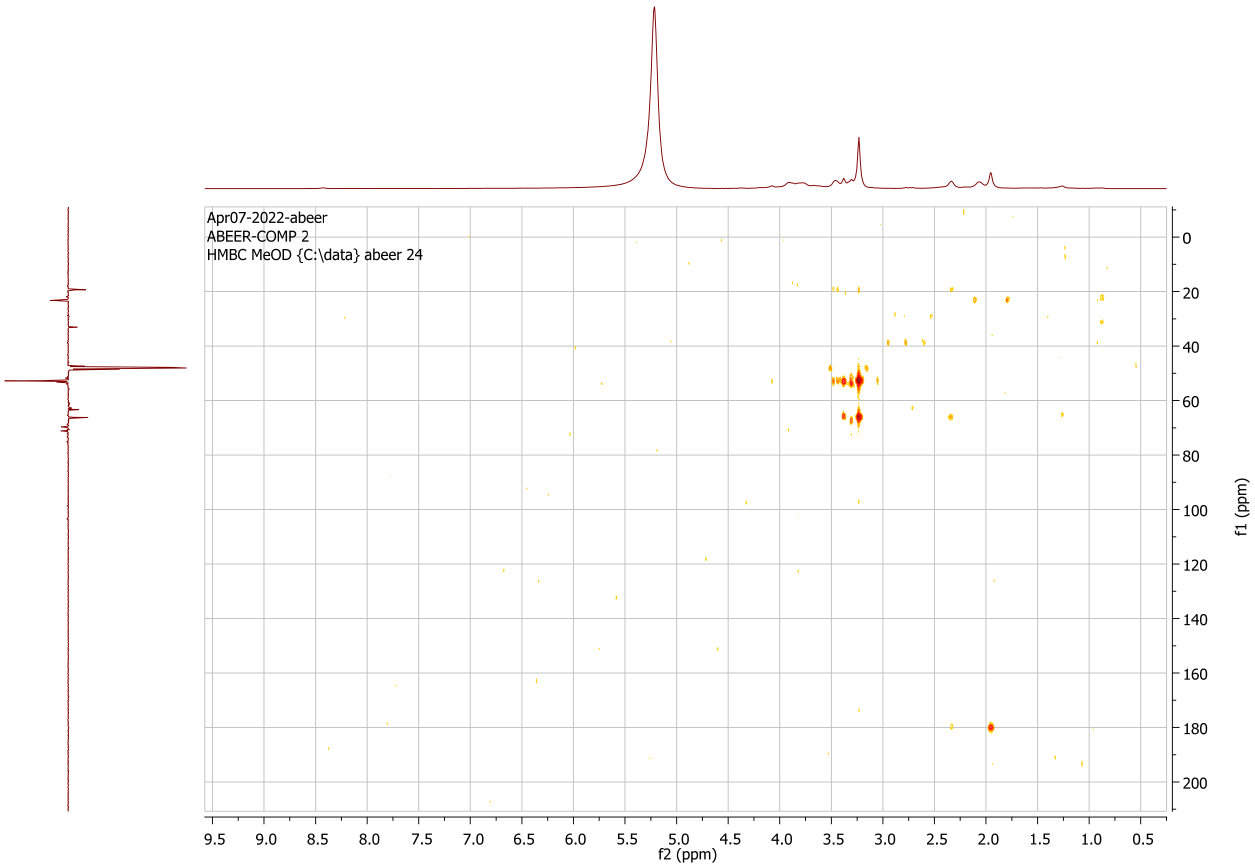

Figure S4. HMBC spectrum of compound **1** measured in CD_3OD-d_4

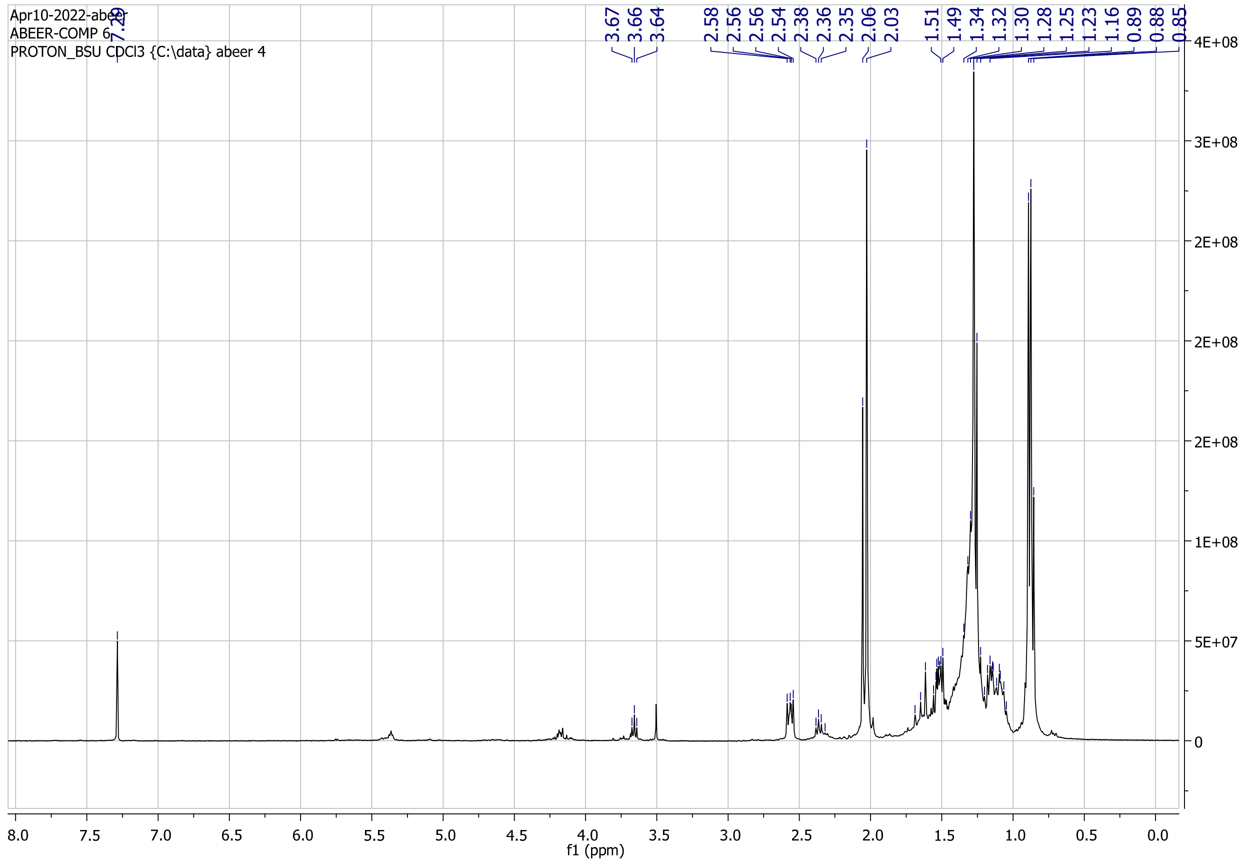

Figure S5. 1H NMR spectrum of compound **2** measured in $CDCL_3$-*d* at 400 MHz

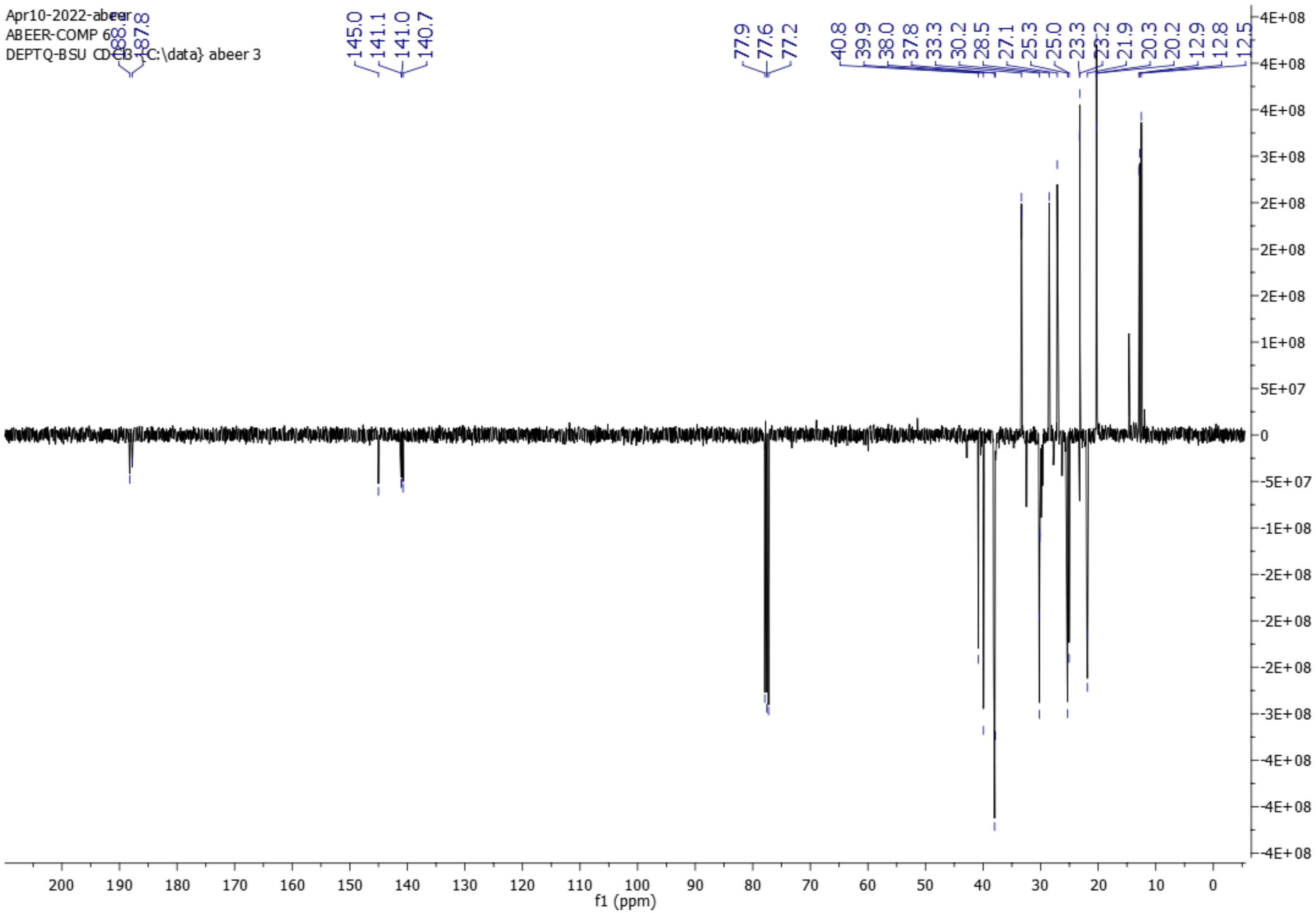

Figure S6. DEPT-Q NMR spectrum of compound **2** measured in $CDCL_3$-*d* at 100 MHz

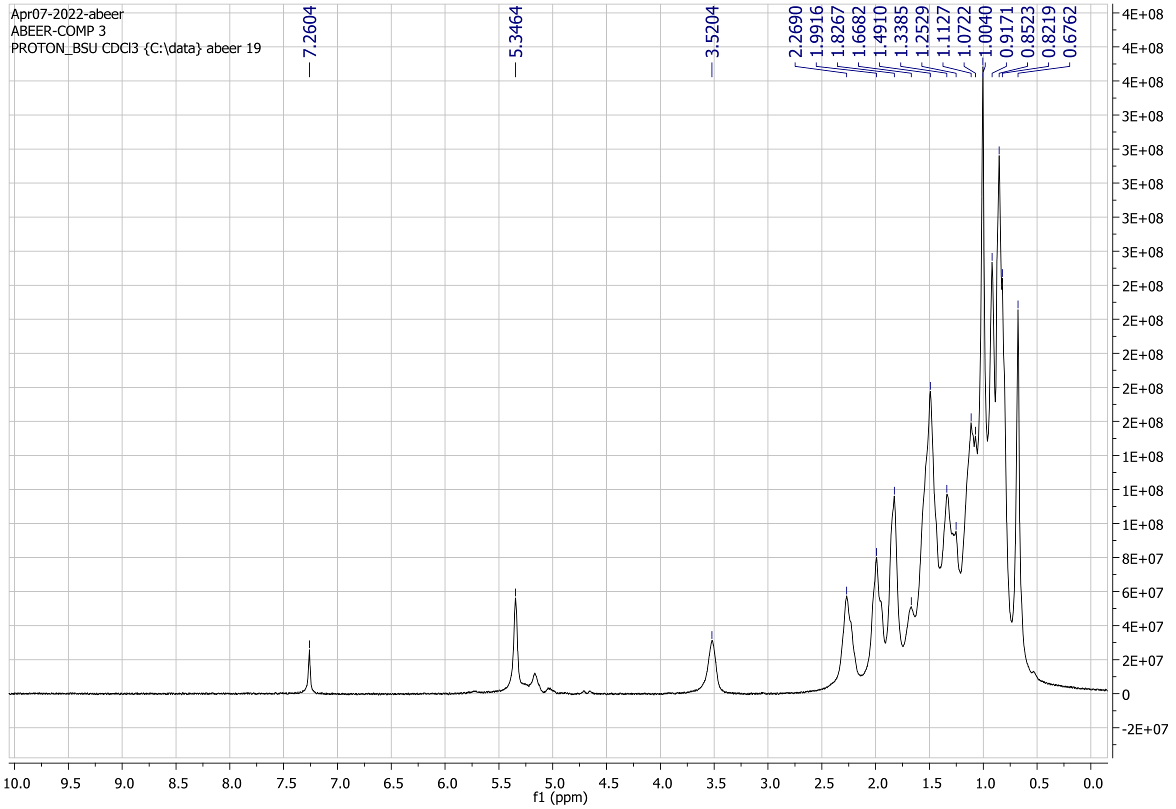

Figure S7. 1H NMR spectrum of compound **3** measured in CDCL$_3$-*d* at 400 MHz

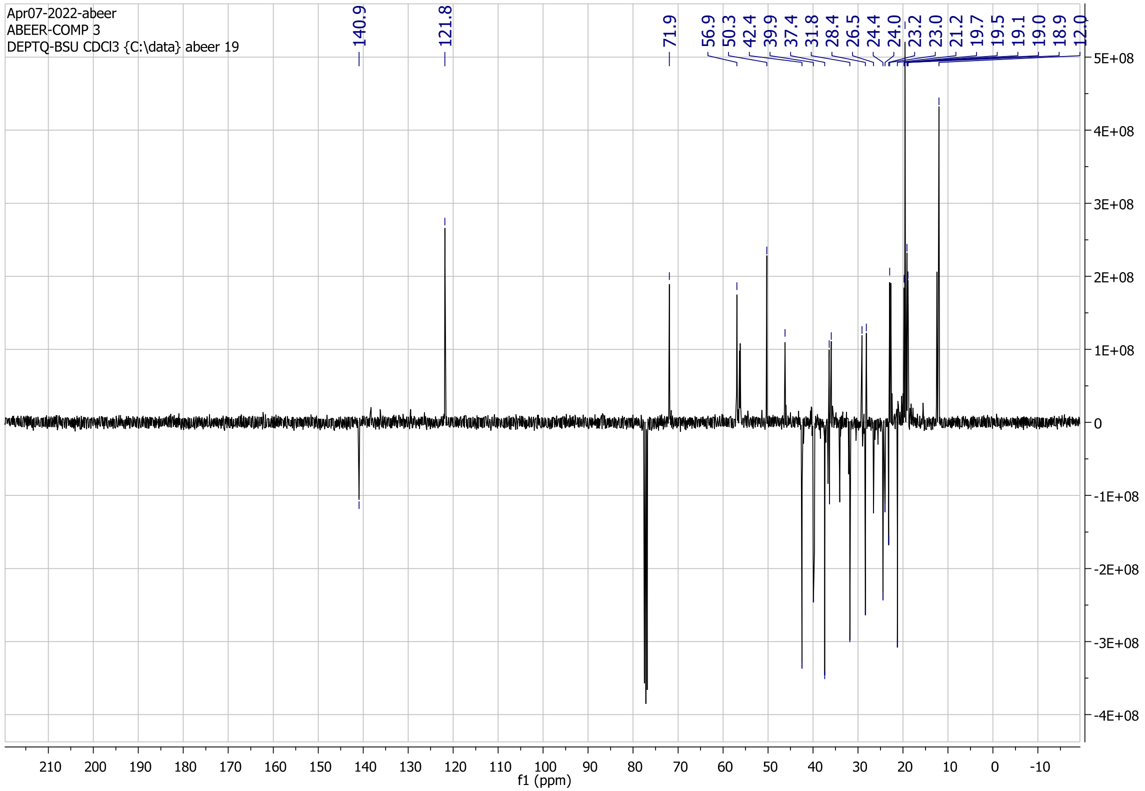

Figure S8. DEPT-Q NMR spectrum of compound **3** measured in $CDCL_3$-*d* at 100 MHz

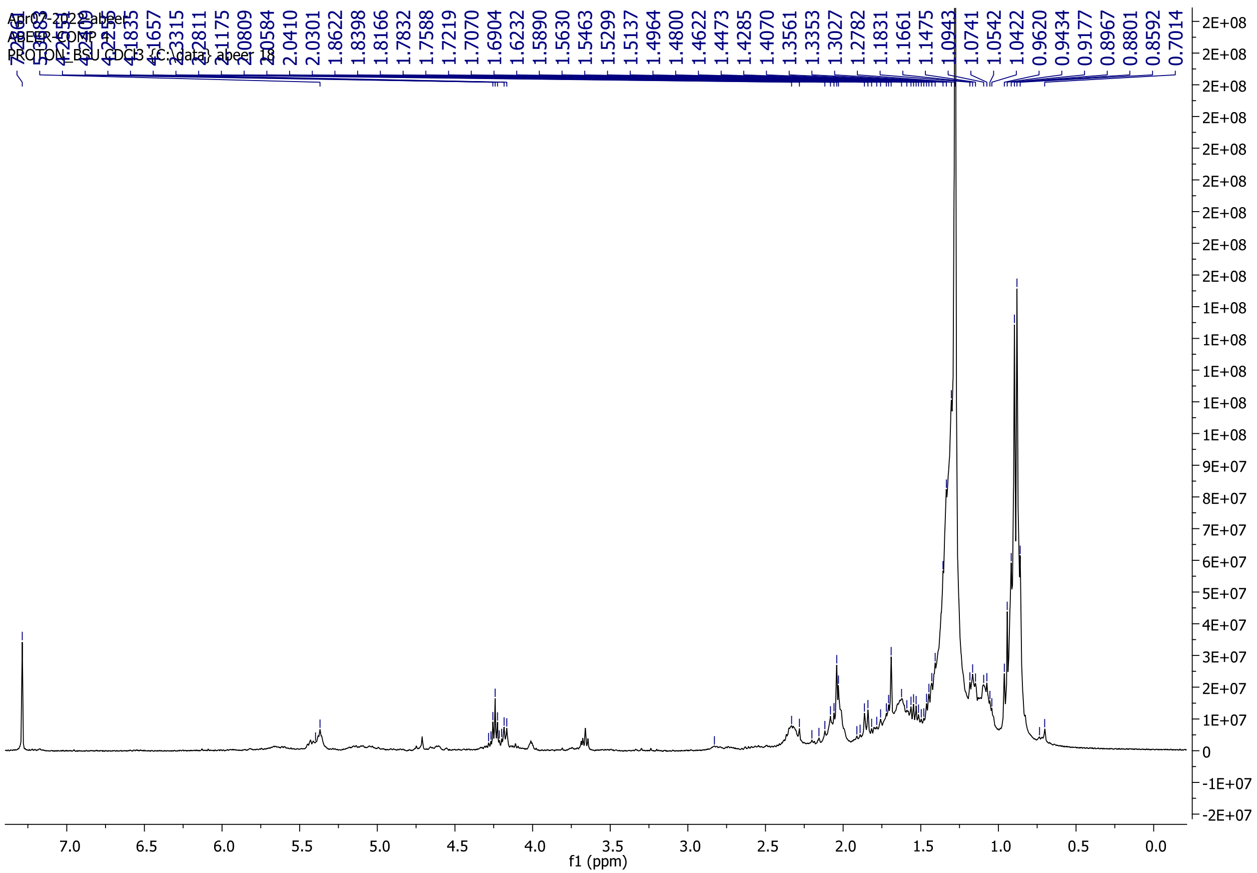

Figure S9. 1H NMR spectrum of compound **4** measured in $CDCL_3$-*d* at 400 MHz

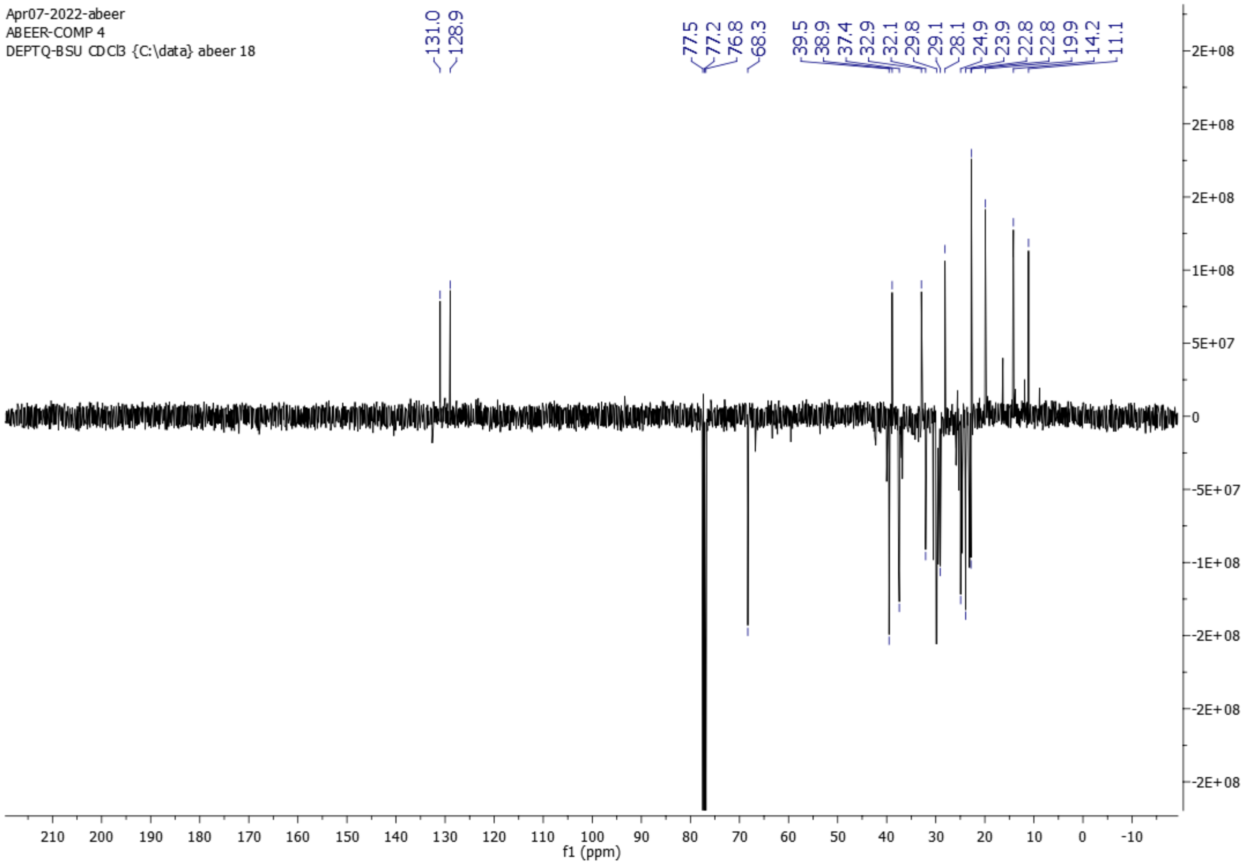

Figure S10. DEPT-Q NMR spectrum of compound **4** measured in $CDCL_3$-*d* at 100 MHz

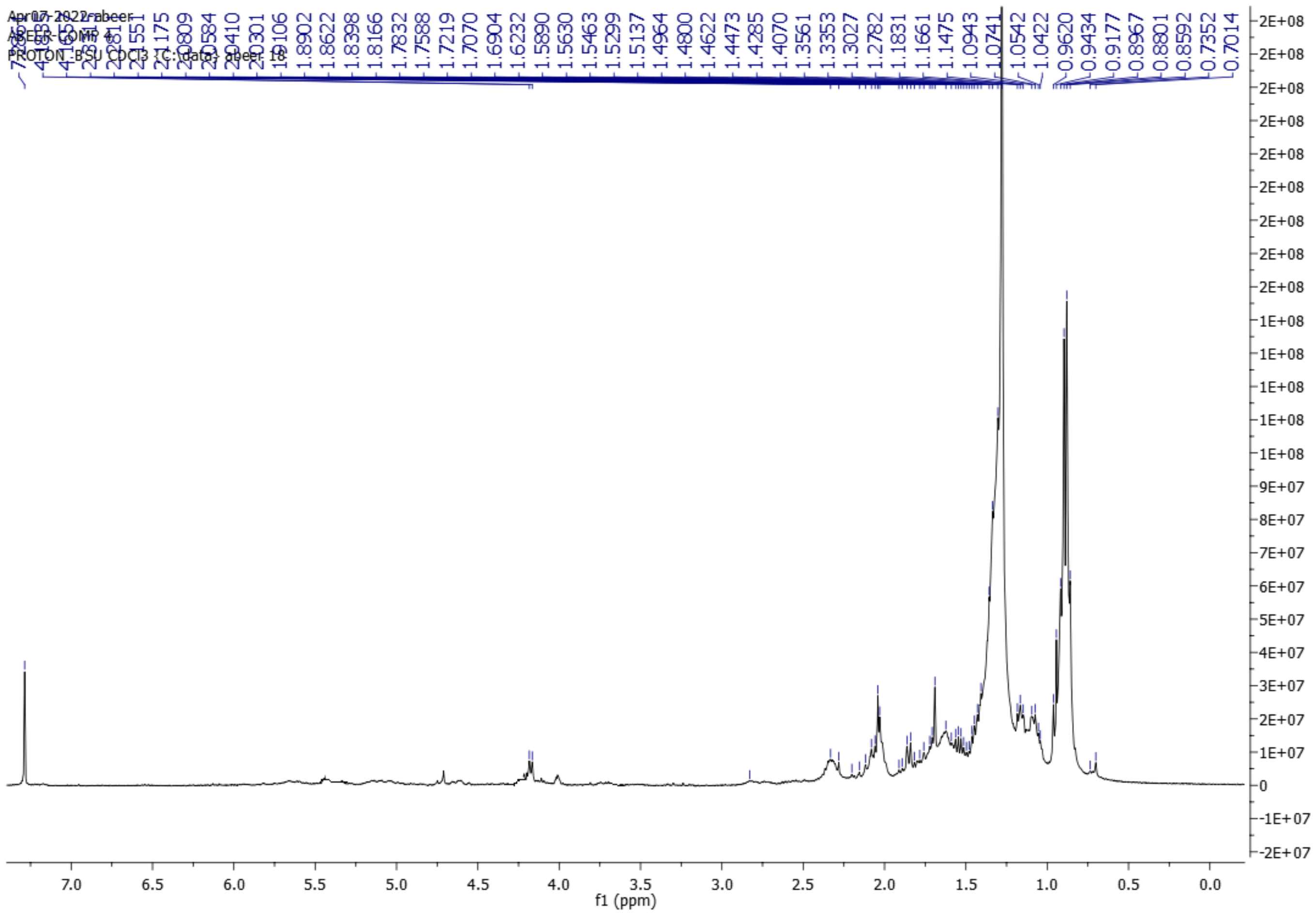

Figure S11. 1H NMR spectrum of compound **5** measured in $CDCL_3$-*d* at 400 MHz

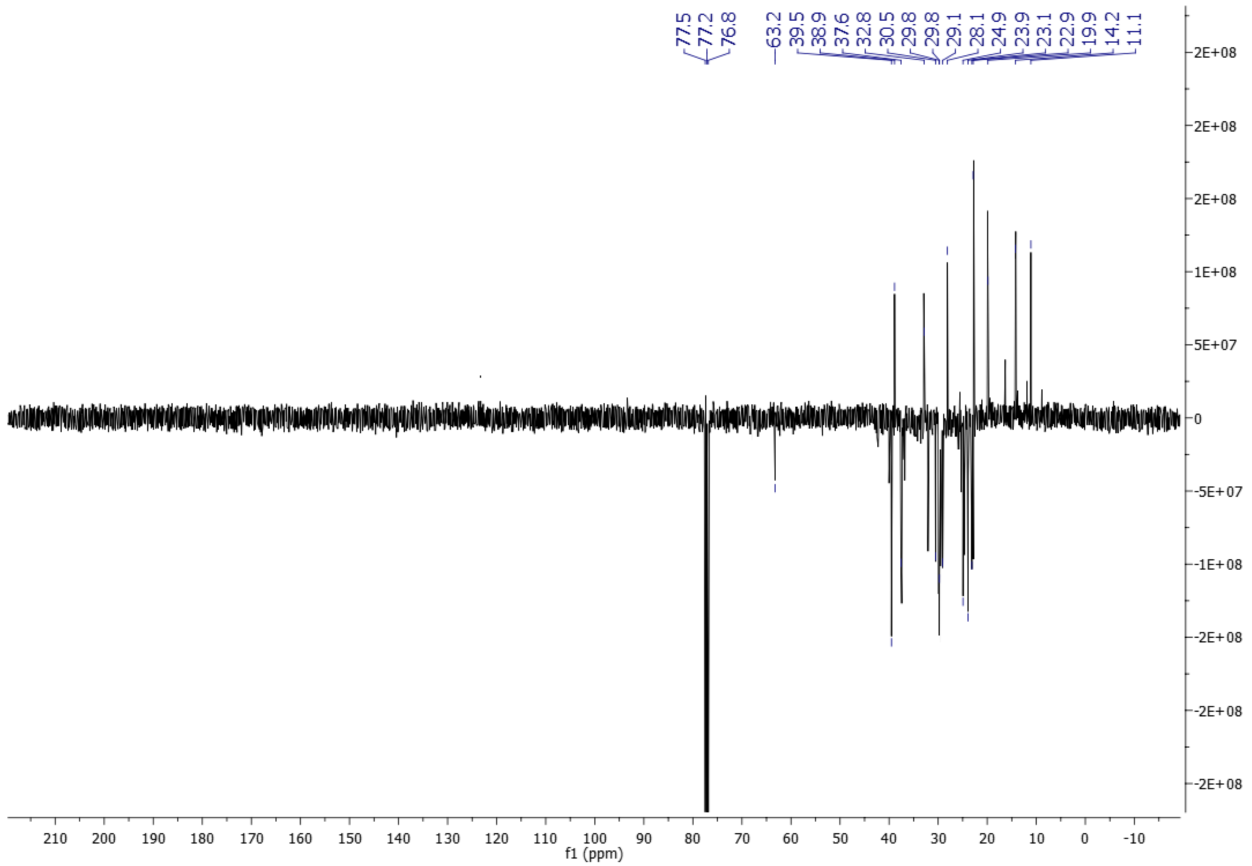

Figure S12. DEPT-Q NMR spectrum of compound **5** measured in $CDCL_3$-*d* at 100 MHz

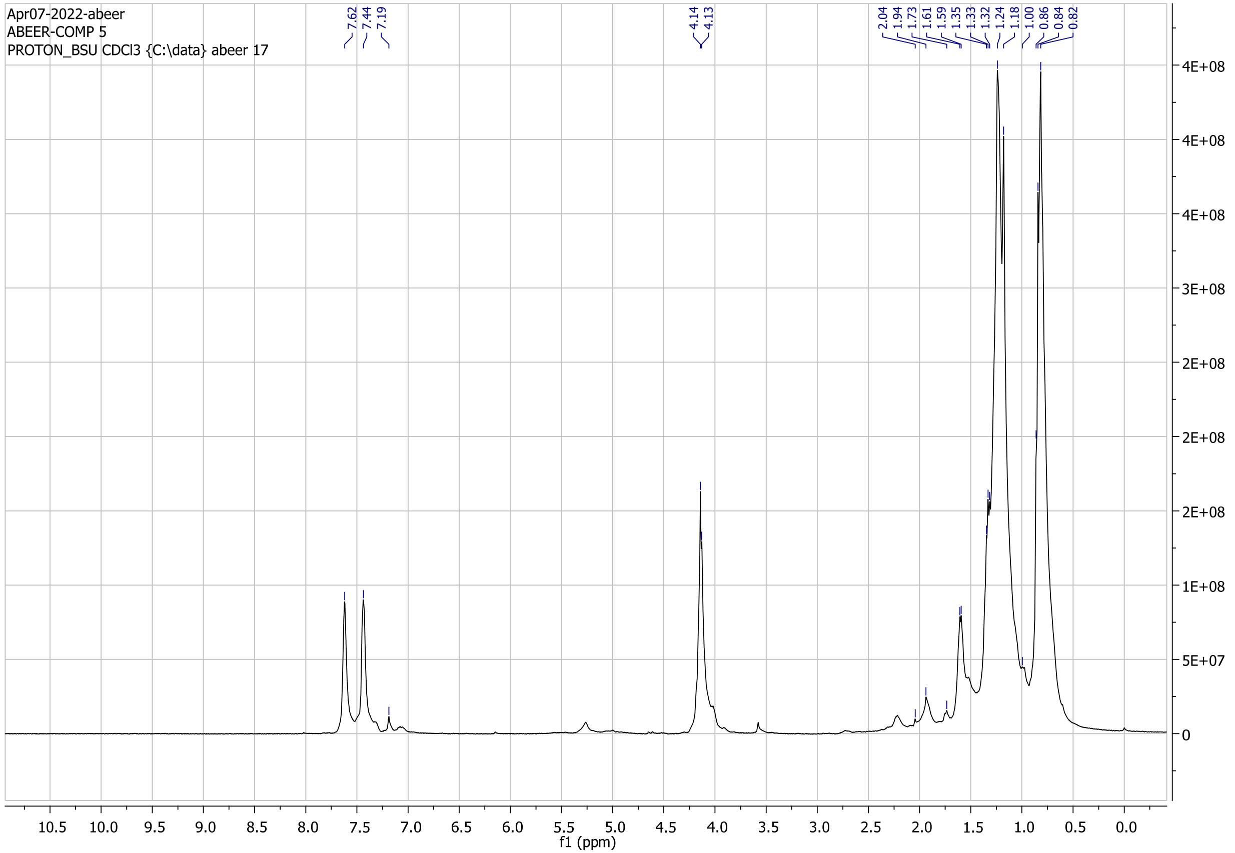

Figure S13. 1H NMR spectrum of compound **6** measured in $CDCL_3$-*d* at 400 MHz

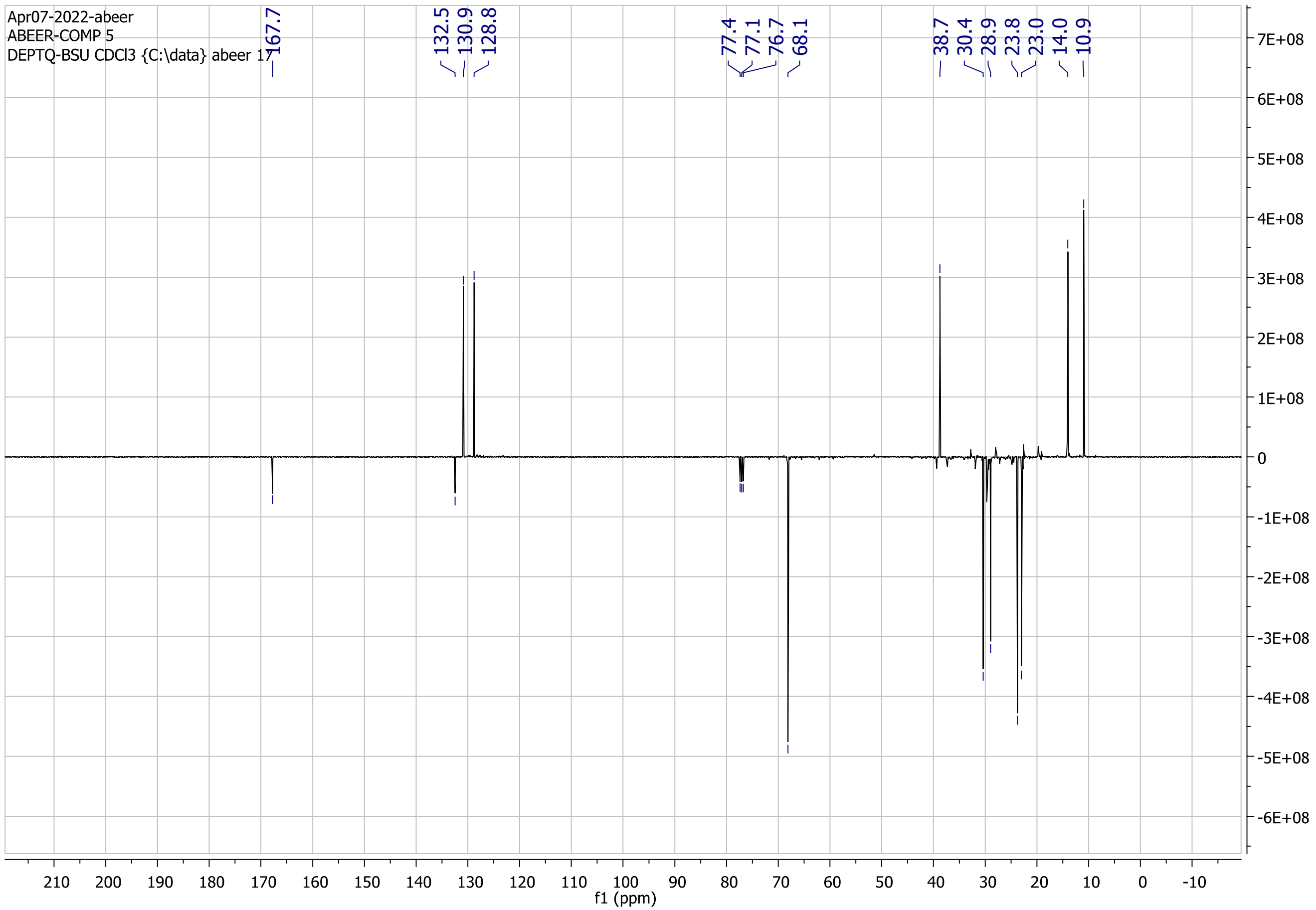

Figure S14. DEPT-Q NMR spectrum of compound **6** measured in $CDCL_3$-*d* at 100 MHz

Methods

Antimalarial assay

Synchronized ring-stage parasites with 1% parasitaemia of *P. falciparum* NF54 strains were plated in triplicate in 96-well plates (200 µL/well) in the presence of a serial dilution of extracts dissolved in 0.5% v/v dimethyl sulfoxide (DMSO). The parasites were incubated with the extracts for 72 h at 37 °C in the presence of 90% N_2, 5% O_2 and 5% CO_2. The incubation of parasites with DMSO at a concentration of 0.5% alone was used as negative control while incubation of parasites with 20% DMSO served as positive control Afterwards, 20 µL was removed and added to 100 µL of the Malstat reagent (1% Triton X-100, 10 mg of l-lactate, 3.3 mg Tris and 0.33 mg of 3-Acetylpyridine adenine dinucleotide dissolved in 1 mL of distilled water, pH 9.0) in a new 96-well microtiter plate. The plasmodial lactate dehydrogenase activity was then assessed by adding a 20 µL mixture of NBT (Nitro Blue Tetrazolium)/Diaphorase (1:1; 1 mg/mL stock each) to the Malstat reaction. The optical densities were measured at 630 nm and the IC_{50} values were calculated from variable-slope sigmoidal dose–response curves using the GraphPad Prism program version 5.

Biological activity predictions using (PASS) software

The neural network-based software Prediction of Activity Spectra for Substances (PASS) [1] (www.way2drug.com) was used for further prioritization of the antimalarial activity of the suggested compounds (1–6). This software can predict > 4000 types of pharmacological and toxicological activities including their mechanism of action, with approximately 85% as acceptable precision, depending on the submitted compound structures that were subsequently screened utilizing the structure–activity relationship database (SARBase). The prediction results were expressed as probability scores (probably active “Pa” or probably inactive “Pi”). These calculated probability scores were determined by linking the structure and functional groups features in the tested molecules that matched or mismatched the specific activities listed in the software-associated database. The higher the Pa values, the more probable the compound to display the suggested pharmacological activity on a scale of 0–1. Pa values higher than 0.5 mean high experimental chance of the suggested pharmacological activity.

Molecular Docking

AutoDock Vina software was used in all molecular docking experiments [2]. All isolated compounds were docked against the Mpro crystal structure (PDB codes: 4PD4) [3]. The binding site was determined according to the enzyme's co-crystallized ligand. The co-ordinates of the grid box were: x = 199.11; y = -26.02; z = 81.26. The size of the grid box was set to be 10 Å. Exhaustiveness was set to be 24. Ten poses were generated for each docking experiment [3,4]. Docking poses were analyzed and visualized using Pymol software [2]. The scores of te generated docking poses alongside the calculated ΔG values are summarized in the following table:

Table 1. Docking scores and binding free energy values of the generated docking scores

**Pose rank**	**Docking score in kcal/mol**	**$\Delta G_{binding}$**
Compound 2		
**1**	-10.83	-8.14
**2**	-10.52	-8.33
**3**	-10.49	-7.98
**4**	-10.44	-7.74
**5**	-10.32	-7.87
**6**	-10.26	-7.53
**7**	-10.23	-7.58
**8**	-10.18	-7.64
**9**	-10.14	-7.46
**10**	-9.94	-7.44

Co-crystalized inhibitor (Atovaquone)		
**1**	-9.76	-14.46
**2**	-9.73	-14.23
**3**	-9.66	-13.48
**4**	-9.58	-13.22
**5**	-9.51	-13.04
**6**	-9.48	-12.86
**7**	-9.42	-13.74
**8**	-9.39	-13.32
**9**	-9.27	-12.54
**10**	-9.23	-13.71

Molecular dynamics Simulation

Desmond v. 2.2 software was used for performing MDS experiments [3,4]. This software applies the OPLS-2005 force field. Protein systems were built using the System Builder option, where the protein structure was checked for any missing hydrogen's, the protonation states of the amino acid residues were set (pH = 7.4), and the co-crystalized water molecules were removed. Thereafter, the whole structure was embedded in an orthorhombic box of TIP3P water together with 0.15 M Na^+ and Cl^- ions in 20 Å solvent buffer. Afterward, the prepared systems were energy minimized and equilibrated for 10 ns. For protein-ligand complexes, the top-scoring poses were used as a starting points for simulation. Desmond software automatically parameterizes inputted ligands during the system building step according to the OPLS force field. For simulations performed by NAMD [5], the protein structures were built and optimized by using

the QwikMD toolkit of the VMD software. The parameters and topologies of the compounds were calculated using the Charmm27 force field with the online software Ligand Reader and Modeler (http://www.charmm-gui. org/?doc=input/ligandrm, accessed on 3 May 2022) [6]. Afterward, the generated parameters and topology files were loaded to VMD to readily read the protein–ligand complexes without errors and then conduct the simulation step.

Absolute Binding Free Energy Calculation

Binding free energy calculations (ΔG) were performed using the free energy perturbation (FEP) method. This method was described in detail in the recent article by Kim and coworkers [7]. Briefly, this method calculates the binding free energy ΔGbinding according to the following equation: $\Delta G_{binding} = \Delta G_{Complex} - \Delta G_{Ligand}$. The value of each ΔG is estimated from a separate simulation using NAMD software. All input files required for simulation by NAMD can be prepared by using the online website Charmm-GUI (https://charmm-gui.org/?doc=input/afes.abinding). Subsequently, we can use these files in NAMD to produce the required simulations using the FEP calculation function in NAMD. The equilibration (5 ns long) was achieved in the NPT ensemble at 300 K and 1 atm (1.01325 bar) with Langevin piston pressure (for “Complex” and “Ligand”) in the presence of the TIP3P water model. Then, 10 ns FEP simulations were performed for each compound, and the last 5 ns of the free energy values was measured for the final free energy values. Finally, the generated trajectories were visualized and analyzed using VMD software.

References

1. Lagunin, A.; Stepanchikova, A.; Filimonov, D.; Poroikov, V. PASS: Prediction of activity spectra for biologically active substances. *Bioinformatics* **2000**, *16*, 747–748.

2. Seeliger, D.; de Groot, B.L. Ligand docking and binding site analysis with PyMOL and Autodock/Vina. *J. Comput. Aided Mol. Des.* **2010**, *24*, 417–422.

3. Bowers, K.J.; Chow, D.E.; Xu, H.; Dror, R.O.; Eastwood, M.P.; Gregersen, B.A.; Klepeis, J.L.; Kolossvary, I.; Moraes, M.A.; Sacerdoti, F.D.; et al. Scalable algorithms for molecular dynamics simulations on commodity clusters. In Proceedings of the SC'06: Proceedings of the 2006 ACM/IEEE Conference on Supercomputing, Tampa, FL, USA, 11–17 November 2006; IEEE: New York, NY, USA, 2006; p. 43.

4. Release, S. *3: Desmond Molecular Dynamics System, DE Shaw Research, New York, NY, 2017*; Maestro-Desmond Interoperability Tools, Schrödinger: New York, NY, USA, 2017.

5. Phillips, J.C.; Braun, R.; Wang, W.; Gumbart, J.; Tajkhorshid, E.; Villa, E.; Chipot, C.; Skeel, R.D.; Kalé, L.; Schulten, K. Scalable molecular dynamics with NAMD. *J. Comput. Chem.* **2005**, *26*, 1781–1802.

6. Kim, S.; Oshima, H.; Zhang, H.; Kern, N.R.; Re, S.; Lee, J.; Rous, B.; Sugita, Y.; Jiang, W.; Im, W. CHARMM-GUI free energy calculator for absolute and relative ligand solvation and binding free energy simulations. *J. Chem. Theory Comput.* **2020**, *16*, 7207–7218.

7. Ngo, S.T.; Tam, N.M.; Quan, P.M.; Nguyen, T.H. Benchmark of Popular Free Energy Approaches Revealing the Inhibitors Binding to SARS-CoV-2 M^{pro}. *J. Chem. Inf. Model.* **2021**, *61*, 2302–2312.
